# Understanding DNA Repair in Hyperthermophilic Archaea: Persistent Gaps and Other Reasons to Focus on the Fork

**DOI:** 10.1155/2015/942605

**Published:** 2015-06-04

**Authors:** Dennis W. Grogan

**Affiliations:** Department of Biological Sciences, University of Cincinnati, 614 Rieveschl Hall, Clifton Court, Cincinnati, OH 45221-0006, USA

## Abstract

Although hyperthermophilic archaea arguably have a great need for efficient DNA repair, they lack members of several DNA repair protein families broadly conserved among bacteria and eukaryotes. Conversely, the putative DNA repair genes that do occur in these archaea often do not generate the expected phenotype when deleted. The prospect that hyperthermophilic archaea have some unique strategies for coping with DNA damage and replication errors has intellectual and technological appeal, but resolving this question will require alternative coping mechanisms to be proposed and tested experimentally. This review evaluates a combination of four enigmatic properties that distinguishes the hyperthermophilic archaea from all other organisms: DNA polymerase stalling at dU, apparent lack of conventional NER, lack of MutSL homologs, and apparent essentiality of homologous recombination proteins. Hypothetical damage-coping strategies that could explain this set of properties may provide new starting points for efforts to define how archaea differ from conventional models of DNA repair and replication fidelity.

## 1. Genome Integrity and Archaea

The importance of maintaining the integrity of cellular genomes, and the diversity of processes that threaten it, can be seen in the network of sophisticated mechanisms that cope with DNA damage and replication errors in even the simplest microorganisms. Consistent with their deep integration into cellular biology, damage-coping strategies show both conservation and divergence; eukaryotes and bacteria employ similar sets of basic coping strategies, for example, but these differ in their mechanistic details. The similarities may reflect the common biological threats posed by DNA lesions and mutations, whereas the differences point to the deep evolutionary divergence between these two fundamentally different types of cells [1].

There are legitimate reasons to expect that archaea, especially the hyperthermophiles, differ from both bacteria and eukaryotes with respect to mechanisms of genome maintenance. As predicted by its distinct evolutionary history [2], the third domain of life shares certain fundamental molecular features only with eukaryotic cells, others only with bacterial cells, and has yet other features that are uniquely archaeal. With respect to genome maintenance, archaea employ “eukaryotic” DNA replication proteins to propagate small, circular chromosomes in the context of a small “prokaryotic” cell [3–7]. In addition, many groups of archaea are adapted to environmental extremes which impose molecular constraints not accommodated by the microorganisms used historically as models of molecular biology. To the extent that archaea may have genetic mechanisms not represented in bacteria or eukaryotes, they represent a potential source of new information concerning early cellular evolution, as well as processes that may be used for biotechnology, especially if these processes occur in the archaea that are adapted to extreme conditions.

Molecular mechanisms not known from other systems are by nature, however, difficult to define, and confirming such mechanisms will require genetics, including the construction and analysis of mutant strains. Because hyperthermophilic archaea (HA) are challenging to manipulate as living cells, their genetic processes have been addressed overwhelmingly* via* biochemical and structural analyses of purified proteins, with little input from the complementary and essential perspective of functional genetics. Paraphrasing a sentiment once expressed by the author's doctoral research advisor, “When you do biochemistry, you tell the organism what you think is important; when you do genetics, the organism tells you what* it* thinks is important.”

## 2. The Functional-Genetic Era for Hyperthermophilic Archaea

For HA, basic genetic analyses have become feasible only in the past decade, and one of the first of these studies targeted the reverse DNA gyrase of* Thermococcus kodakaraensis* [8]. Reverse gyrases (Rgy) are single-strand-nicking DNA topoisomerases that introduce positive supercoils into DNA and appear only in extreme- or hyperthermophilic archaea and bacteria [9]. Although its natural distribution suggested to many that reverse gyrase activity is essential for life at high temperature, Atomi et al. [8] successfully isolated a deletion mutant, demonstrating that Rgy is not essential for* Thermococcus* viability. The* Thermococcus* mutant nevertheless grew at a reduced rate and with a decreased maximal temperature. Thus, whereas Rgy appears not to be absolutely required for cellular function in* Thermococcus*, the mutant phenotype argued that it must play a role of some importance. The latter conclusion has been reinforced by failure of later efforts to delete the corresponding genes of other hyperthermophilic archaea [10].

In the ensuing decade, progress in the genetic analysis of DNA metabolic enzymes of HA has been substantial but hard-won. As in the Rgy study, the archaeal mutants are typically constructed by replacing the target gene with a selected marker and therefore represent unambiguous loss of gene function. However, the resulting genotype often does not match that predicted by the bacterial or eukaryotic counterpart. On one hand, this reinforces the evidence of deep divergence separating archaea from bacteria and eukaryotes and the incentive for their analysis, but on the other hand, it intensifies the need to formulate and test the next generation of hypotheses. This review and commentary selectively interprets functional genetic data relating to genome stability in archaea, with an emphasis on the hyperthermophiles. It proposes that certain patterns of results do not fit well with the schemes of genome-maintenance mechanisms identified in eukaryotic or bacterial models and suggests ways in which atypical or primordial damage-coping strategies could, in principle, support fundamentally different strategies of genome maintenance in the HA.

## 3. Mutant Phenotypes

Table 1 lists functional studies of HA genes that have been implicated in DNA repair or related processes (usually by sequence similarity), grouped according to the phenotypic outcome that was observed. Mutants placed in Group I seem to be relatively straightforward to interpret, since their phenotypes indicate a role of the deleted gene in DNA repair or mutagenesis roughly in line with that predicted from its sequence. Examples include DNA photolyases, at least one TLS polymerase, Holliday-junction resolvases, a 3′-flap endonuclease, a recombinase homolog, and two putative uracil DNA glycosylases (UDG). In most cases, the documented phenotype is increased sensitivity to one or more DNA-damaging agents, although the UDG mutants showed impaired growth [10] and the Y-family polymerase mutant showed an altered spectrum of spontaneous mutation [11].

Mutants in Group II identify genes with minimal impact on DNA repair capacity, as evidenced by little or no detectable increase in sensitivity to radiation or DNA-damaging chemicals. In some cases, notably HJ resolvases, Hjc and Hje, single mutants exhibited only marginal phenotypes, but the double mutant was apparently nonviable [12]. The latter case thus suggests that some cellular function of these two enzymes is important and is fulfilled by two at least partially redundant enzymes. An important feature of the Group II cases (Table 1) is that most of them encode homologs of eukaryotic NER proteins, which were expected to participate in generalized repair of bulky, helix-distorting DNA lesions. Although redundancy of function could, in principle, account for this result, the corresponding eukaryotic genes typically do not exhibit such redundancy with respect to DNA repair [13].

Group III of Table 1 represents genes which could not be deleted and thus may encode an essential function. Although the genetic tools for HA remain limited, in some cases lethality could be further supported by specific genetic tests. Some of the proteins are predicted to play important roles in basic replication, and thus their appearance in this group is fully plausible; examples include the structure-specific endonuclease Fen1, which is implicated in maturation of Okazaki fragments. Most of the genes in this category are associated specifically with homologous recombination, which is however not an essential process in normal cells of bacteria, unicellular eukaryotes, or mesophilic archaea.

As noted in several of these studies, the number of putative DNA-repair genes that fall into the latter two categories (Groups II and III) adds to the accumulating evidence that HA do not follow the bacterial or eukaryotic models of DNA repair in any strict sense. The available evidence (discussed below) nevertheless argues that they do replicate their genomes accurately and cope effectively with DNA damage. This poses the interesting but challenging opportunity of identifying precisely how the genome-maintenance mechanisms of HA differ from those defined historically in bacteria and eukaryotic cells. This review and commentary highlights four* enigmata* of genome-stability mechanisms in HA, some of which have persisted for two decades: (i) the functional role of archaeal DNA polymerase stalling at dU, (ii) the apparent lack of repair-specific functions for the HA homologs of eukaryotic NER proteins, (iii) the specific absence of MutSL homologs in HA, and (iv) the essentiality of HR proteins in HA. Although the interpretations provided here are broad and speculative, attention to unresolved questions such as these represents a logical prerequisite to uncovering genetic processes that distinguish archaea, and especially HA, from other forms of life.

## 4. The Enigma of DNA Polymerases Blocked by Template dU

Although early characterization of HA DNA polymerases showed that they have certain advantages for PCR relative to Taq DNA polymerase, it was also found that HA polymerases bind tightly to any dU they encounter in the template strand [14], which impedes PCR. Driven by the practical incentives to use HA DNA polymerases in PCR, various biochemical techniques have been developed to overcome this problem. In addition to enabling PCR by HA DNA polymerases, these techniques illustrate the potential biological challenges posed by this uniquely archaeal property of these polymerases.

One technique which promotes PCR by archaeal polymerases involves supplementing the reaction mixture with a thermostable uracil DNA glycosylase (UDG) [15]. Because UDG destroys the dU-containing strand as a usable template in any subsequent cycle, its beneficial impact implies that dU binding does not merely slow the HA polymerase but somehow removes a significant fraction of it from contributing to DNA synthesis. This suggests that the polymerase:dU complex is both unproductive and stable and raises questions about what impact such properties would have* in vivo*.

Another technique involves supplementing the reaction mixture with a thermostable dUTPase [16]. This enzyme removes dUTP from the cellular pool of dNTPs, thus preventing its incorporation into nascent DNA* in vivo.* The impact of this technique suggests that much of the template dU that the polymerases of HA encounter during PCR results from cytosine deaminated before, not after, it was incorporated into DNA. This is consistent with* in vitro* studies demonstrating limited discrimination against dUTP by DNA polymerases of HA [17] and raises the biologically relevant question as to how well HA purge dUTP from their nucleotide pools during chromosome replication. A third approach to improved PCR by these polymerases has been to mutate the specific dU-binding pocket which mediates the stalling. This has yielded fully active and thermostable archaeal DNA polymerases which are nevertheless insensitive to the occurrence of dU in the template strand [18, 19].

The only biological rationale offered consistently in the literature for the specific stalling of the replicative polymerases of HA at template dU is to avoid G:C to A:T transition mutations. The logic is admittedly simple and plausible: (i) hydrolytic deamination of dC occurs spontaneously in DNA and is greatly accelerated by high temperature and (ii) most DNA polymerases (including those of HA) insert dA opposite the resulting dU, thus creating a transition mutation. Although avoiding such mutation is clearly a benefit, the magnitude of this benefit has not been weighed against the risks and costs which polymerase stalling imposes on the archaeal cell nor against the benefits of conventional repair, nor has it been evaluated critically in the wider context of DNA metabolism.

For example, why does the property of polymerase stalling at dU correlate with phylogenetic domain, not with growth temperature? All the replicative DNA polymerases of archaea that have been examined, but none of the eukaryotic or bacterial enzymes, stall at dU [20]. This natural distribution thus seems to undermine the argument that HA need polymerase stalling specifically to cope with a high rate of spontaneous cytosine deamination in DNA, as hyperthermophilic bacteria should have a similar need, whereas mesophilic archaea should not. It also seems striking that D-family DNA polymerases, which replicate genomes and occur only in archaea, are also inhibited by dU, but through a different mechanism [21]. Moreover, there seems to be (at least superficially) no evidence that HA are any less efficient in the conventional base-excision repair (BER) of dU than thermophilic bacteria are. Corresponding N-glycosylases, supported by AP lyases, AP endonucleases, and related BER enzymes, are well represented in HA [22].

**Figure 1:**
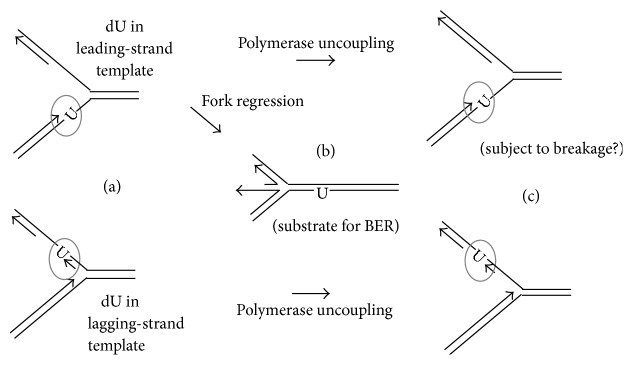
Possible fates of a replicative polymerase stalled at template dU. (a) Archaeal polymerases stall with the nascent 3′ end 4 nt ahead of the template dU [23]. (b) If the replication fork responds with immediate fork reversal, the dU is restored to its original context, and BER is required for resumption of fork progress. (c) If tight coupling is not preserved, the Mcm helicase and nonstalled polymerase (not drawn) would continue, creating ssDNA region on the stalled template vulnerable to structure- or single-strand-specific nucleases. Arrowheads on DNA strands represent 3′ ends.

Other, more mechanistic, questions concern the fact that the stalled DNA polymerase precludes BER, by sequestering the dU within the binding pocket of the polymerase [23]. Repair thus requires some form of replication-fork reversal or disassembly. How this process might work remains difficult to test. The apparently most favorable and commonly proposed outcome is reversal of the fork, allowing BER of the dU in its original context (Figure 1). Recently, an archaeal DNA primase has been found to bypass dU and other damaged bases* in vitro* [24], and this may allow bypass with minimal reversal or rearrangement of the fork. Other plausible scenarios can be proposed which have not been discussed in the literature; these involve intermediate structures that are vulnerable to single-strand or structure-specific nucleases, leading potentially to breakage (or “collapse”) of the replication fork (Figure 1). As argued below, fork reversal, disassembly, or even breakage may be a routine process in HA chromosome replication and may underlie uniquely archaeal strategies to repair DNA lesions. Even in this context, however, the antimutagenesis argument fails to identify the net advantage for archaea to invest deeply in a radical alternative to the relatively simple, low-risk strategy of BER, which these archaea also employ.

More evidence of deeper complexity in the dU-stalling phenomenon comes from recent gene-deletion studies. Genome annotations depict* S. islandicus* as having ensured an adequate level of dU repair, as it encodes no fewer than four putative UDG proteins. However, efforts to delete each of the genes individually were successful in only two cases, and the successful constructs showed impaired growth under laboratory conditions [10]. Since neither lethality nor growth impairment is a characteristic of UDG mutants, even of similarly thermophilic bacteria [25], these results argue that the encoded* Sulfolobus* proteins have roles beyond the basic, redundant BER of dU predicted by their annotation as four UDGs.

## 5. Do Archaea Need dU for a Specific Aspect of Normal Chromosome Replication?

As noted above, deamination of dC is not the only source of dU; it can also be inserted opposite dA via dUTP incorporation during polymerization. The dU formed by this route is not intrinsically mutagenic, and its abundance would be determined by the discriminative accuracy of the DNA polymerase against dUTP [17] and the level of dUTP in the cellular pools. To the author's knowledge, neither dUTP levels nor rates of dUTP incorporation have been measured in archaeal cells.

Removal of dU from archaeal DNA is expected to occur at a steady rate dictated largely by intracellular levels of UDG. In contrast, dU incorporation occurs only at sites of DNA synthesis; dU would thus be most abundant immediately after passage of the replication fork and would decay steadily over time thereafter. The contrasting kinetics of incorporation versus removal could, in principle, make dU a “biochemical signature of youth,” marking strands that remain uncorrected by BER because they have been synthesized recently. It is tempting to question whether this or some other molecular “time stamp” could control important features of genome replication in archaea.* E. coli* uses methylation of the tetranucleotide GATC in an analogous way to distinguish old and new strands after the replication fork has passed, and this signal is necessary for the accuracy of postreplicative MMR [26–28]. In HA, dU could, in principle, play an analogous role in a uniquely archaeal error-correction system, or it could serve to limit the extent or timing of DNA synthesis in recently replicated regions. Alternatively, optimal replication in HA may simply require periodic fork reversal in the absence of replication-blocking lesions, for which dU in the template strand may provide a specific trigger.

Whatever cellular processes dU abundance may control, the “biochemical marker” hypothesis predicts that a particular balance of the relevant biochemical activities (dUTPase, UDG, and polymerase stalling) may be important for normal cellular function. Thus, artificial manipulation of these activities may perturb normal replication or growth, which may explain the unexpected results of UDG gene deletion in* S. islandicus* [10]. It may be feasible to test this more directly by manipulating intracellular levels of dUTPase and of individual UDGs through cloning in appropriate expression vectors [29]. It also may be possible to express physiological levels of the recently engineered dU-insensitive DNA polymerases [18, 19] in HA cells using similar vectors.

## 6. The Enigma of Unemployed Eukaryotic NER Proteins

Historically, the molecular dissection of DNA repair pathways, including that of nucleotide excision repair (NER), depended heavily on the nonessential nature of these functions in microorganisms, which allowed completely repair-defective mutants to be isolated and characterized. NER is one of the most generalized systems of DNA repair; its substrates represent a broad spectrum of lesions that are bulky, helix-distorting, or both. Despite having broadly similar strategies, bacterial and eukaryotic NER are mediated by proteins that share little homology. Furthermore, major groups of eukaryotes diverge with respect to the damage recognition proteins which initiate NER; yeast has one set of damage-recognition proteins, plant cells have a different set, and mammalian cells have several additional proteins [13, 30]. In addition to being diverse, the damage-recognition proteins are also the only NER-specific proteins of eukaryotes, as all “downstream” NER proteins play other roles in the cell. The overall pattern thus suggests an evolutionary scenario in which NER arose rather recently and multiple times, by recruitment of suitable proteins to perform the damage-recognition step.

Superficially, genome annotations of mesophilic archaea suggest a redundancy of NER systems in which an incomplete set of eukaryotic NER genes, commonly annotated as “XP” (*xeroderma pigmentosum*) homologs, is superimposed on a complete set of bacterial genes, represented by homologs of* E. coli uvrABC*. None of the archaeal genomes encodes a clear homolog of an eukaryotic NER damage-recognition protein, and the available experimental evidence suggests that only the Uvr proteins mediate NER in these archaea. For example, the damaged oligonucleotide excised by the moderately thermophilic* Methanobacterium thermoautotrophicum* (i.e.,* Methanothermobacter thermoautotrophicus*) strain ΔH (which has a complete set of* uvr* genes) has the length characteristic of bacterial NER, not eukaryotic NER [31]. Similarly, in the mesophilic halophile* Halobacterium salinarum*, inactivation of any* uvr* homolog is sufficient to render the cells UV-sensitive [32]. Thus, the redundancy of NER in mesophilic archaea suggested by genome analysis is more apparent than real, as these archaea seem to rely functionally on the simpler NER system they share with bacteria [33].

This conclusion raises questions for the HA, which uniformly lack the UvrABC homologs found in the mesophilic archaea. Three possibilities can be considered: (i) in HA, lack of the UvrABC system is compensated by recruitment of unidentified, HA-specific, damage-recognition proteins which, in combination with the archaeal XP homologs, reconstruct an eukaryotic-style NER, (ii) HA use fundamentally different “alternative excision” pathways [1] to repair duplex DNA between rounds of replication, or (iii) HA simply forgo preemptive repair and deal with bulky, helix-distorting DNA lesions after they stall the replication fork.

The accumulating experimental evidence argues increasingly against (i), as deletions of most archaeal XP homologs have negligible effects on the survival of UV and other relevant DNA damage (Table 1). While it could be argued that the genes which showed no apparent role in repair duplicate the function of another gene, such redundancy is not seen typically in bacteria and eukaryotes. It should also be noted that HA can remove such lesions and do not merely bypass them, as UV photoproducts are steadily lost from the genomic DNA of UV-irradiated cultures upon resumption of incubation [34, 35]. In addition, the removal of UV photoproducts is not affected by the transcription of the damaged DNA, which argues further that HA do not employ the archaeal RNA polymerase extensively as a DNA damage detector [34, 35].

## 7. Do HA Use DNA Replication Itself to Target Bulky, Helix-Distorting Lesions for Removal?

**Figure 2:**
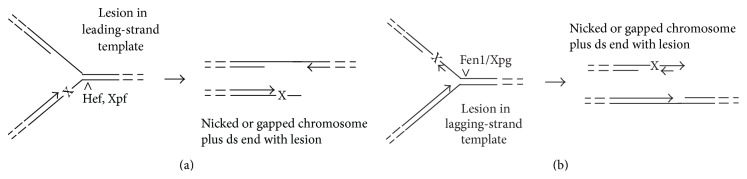
Fork breakage “collapse” in response to replication-blocking lesions. Adducts or other large, helix-distorting lesions (symbolized by X) block a polymerase molecule, inducing a certain degree of uncoupling and ssDNA exposure at the base of the fork. Cleavage by an appropriate single-strand-specific or structure-specific endonuclease can, for each case, generate dsDNA end that retains the lesion. The top strand in each DNA duplex is oriented with the 5′ end on left; free 3′ ends are represented by arrowheads, whereas broken lines indicate the rest of the chromosome. Carets (∧) locate the single cut that would be made by the indicated nuclease. For the cleavage preferences of archaeal Hef, Xpf, and Fen1/Xpg, see [36, 65, 66].

Other experimental evidence can be interpreted as supporting (iii) above. Among various HA deletion mutants, for example, one of the few that fits the phenotype expected of a generalized excision repair deficiency is the Δ*hef* mutant of* Thermococcus kodakarensis* [36]. The encoded Hef protein is related to eukaryotic FANCM proteins and, like them, binds to branched DNAs that mimic stalled replication forks, where it cleaves the duplex near the joint [36]. This specificity seems tailored to the expected structures of archaeal replication forks stalled by unrepaired bulky adducts, particularly if the replicative helicase, archaeal Mcm, mimics the eukaryotic Mcm in its ability to progress past most DNA lesions, leaving them to stall the polymerase [37]. If the leading-strand polymerase encounters the lesion, its blockage would create a partially single-stranded region near the junction of the fork (Figure 2). Cleavage at the base of the fork by Hef or a 3′-flap endonuclease such as archaeal Xpf, or cleavage of the large single-stranded region by ssDNA endonuclease, would break the fork in a way that leaves the lesion on the resulting end. Conversely, a lesion encountered on the lagging-strand template would yield a similar result if the Y-structure were cleaved by a 5′-flap endonuclease, such as Fen1/Xpg (Figure 2). The logic of the proposed strand specificity is that in both cases it positions the lesion for removal by ds end-processing, which is central to replication fork reassembly via HR (discussed in more detail below). In contrast, models of replication-fork “collapse” and reassembly in bacteria have historically proposed steps that leave the lesion in a continuous (albeit potentially gap-containing) DNA; this feature may reflect the rationale that providing additional chances for conventional NER represents the best strategy for successful replication [36, 38–40].

Although inducing fork disassembly and breakage in order to expose and remove DNA lesions seems drastic (as argued above for dU), the lesions in question are, by definition, intrinsically incompatible with replicative polymerases and (unlike dU) may have no alternative pathways of removal in HA. A strategy like this may represent one of the simplest and earliest responses to DNA damage, and its basic feasibility is demonstrated by the relatively robust growth of bacterial and eukaryotic NER mutants under laboratory cultivation. In these cells, the low level of bulky lesions that form endogenously (e.g., protein:DNA cross-links) is usually handled at the replication fork by HR-dependent processes, such as fork reassembly initiated by fork breakage (discussed below), template-switching, or lesion-skipping processes [41].

## 8. The Enigma of Accurate Replication without MutSL Homologs

Researchers analyzing the first* Pyrobaculum* genome sequences noted that these HA genomes did not encode any homologs of the* E. coli* MutS or MutL proteins [42]. These two proteins cooperate to remove replication errors from dsDNA soon after the bacterial replication fork has passed (sometimes termed the “spellchecker” function); they also trigger the abortion of HR between sequences that differ at multiple sites (termed the “antirecombination” function) [26, 27]. Among archaea, the absence of MutSL homologs applies specifically to hyperthermophiles and thus parallels the absence of Uvr proteins. Also similar to Uvr, absence of MutSL homologs in HA does not correlate with any obvious defect in maintaining genome integrity, although it must be noted that few HA have been analyzed in this respect.* Sulfolobus* spp. have been found to replicate their chromosomes accurately, although in some species this may be masked by high levels of transposable element activity [43–45]. The error rate per genome replication is lower in* S. acidocaldarius* than in nearly all other microbial genomes similarly analyzed; interestingly, however, single-nucleotide frameshifts in short mononucleotide runs are prominent in the spectrum, which is a qualitative characteristic of MMR-deficient cells [44].

These results demonstrate that* Sulfolobus* spp. imitate the results of conventional MMR quantitatively, but perhaps not qualitatively. The absence of any MutSL pair argues that* Sulfolobus* spp. and other HA either (i) lack any form of postreplicational mismatch repair or (ii) employ some alternative to the conventional (i.e., MutSL-dependent) strategy. In order to explain the low error rate, hypothesis (i) would require HA to have a much higher accuracy of polymerization* in vivo* than that exhibited by known replicative DNA polymerases* in vitro*. Similarly, (ii) would require some system that recognizes mismatches after the duplex leaves the polymerase and removes the newly synthesized strand but does not involve a MutS or MutL protein.

Explaining the accurate chromosome replication in* Sulfolobus* spp. must also account for their multiple translesion synthesis (TLS) DNA polymerases, which are nonprocessive but error-prone. The* Sulfolobus* Y-family polymerases Dpo4 (*S. solfataricus*) and Dbh (*S. acidocaldarius*) have been cocrystallized with many DNA substrates, providing unprecedented resolution of biochemical and structural analysis of TLS* in vitro* [46]. Both polymerases are highly error-prone when replicating intact template* in vitro*, with Dbh making 1-nt deletions at frequencies over 50% opposite common sequence motifs, such as 5′-GYY- [47]. However, the genetic consequences of inactivating Dbh indicate that this polymerase plays at least one antimutagenic role* in vivo*, namely, the suppression of spontaneous G:C to T:A transversions [11]. The hypothesis that this suppression reflects accurate bypass of oxidized guanine (oxoG) is supported by analysis of individual TLS events past this damaged base (Sakofsky & Grogan, unpublished). However, the most common spontaneous mutations observed in the* S. acidocaldarius* chromosome are not affected by inactivating Dbh (Sakofsky & Grogan, unpublished). Given the extreme inaccuracy of this DNA polymerase* in vitro*, these results suggest that Dbh is normally excluded from the* Sulfolobus* replication fork by an unidentified, yet apparently effective, mechanism.

## 9. Mismatch Processing in* Sulfolobus*

**Figure 3:**
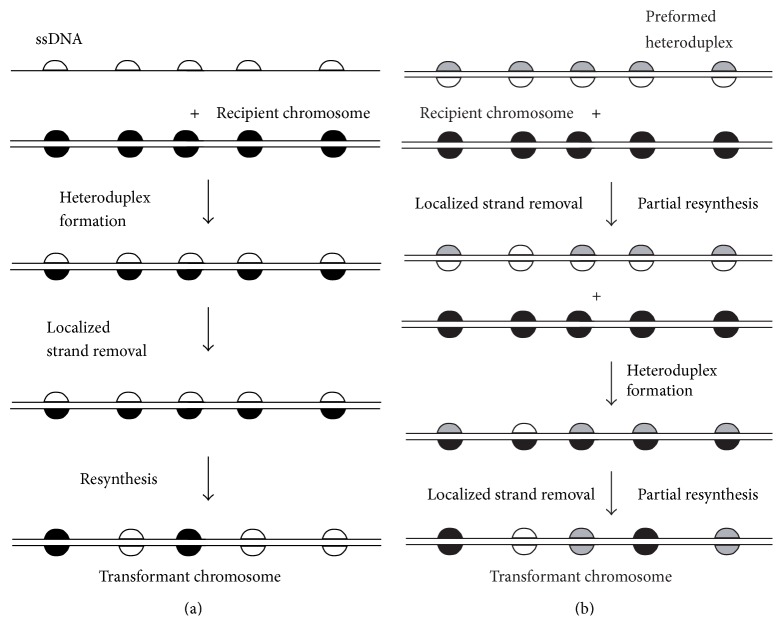
Patchy recombination in* Sulfolobus*. Cells transformed with linear DNAs marked at multiple sites (or mated with multiply marked donor cells) produce recombinants that indicate erratic, localized strand loss from heteroduplex intermediates [48, 67]. (a) Markers are acquired from ssDNA as multiple short tracts, some of which consist of a single marker. (b) A preformed heteroduplex, containing two distinct donor alleles at each marked position, generates similar short patches representing all three possible alleles. Semicircular symbols depict “silent” genetic markers (synonymous mutations), and the colors (black, white, and gray) depict different alleles. Different colors opposite to each other indicate a mismatch that is eventually resolved by strand removal and resynthesis.

Processing of mismatched DNA has been detected genetically in* S. acidocaldarius*, but in the context of genetic recombination, not that of replication accuracy. The evidence comes from transforming auxotrophic mutants with linear DNAs that contain several closely spaced, phenotypically silent base pair substitutions (BPSs) as genetic markers [48]. The complex pattern of markers recovered after transformation with ssDNA (Figure 3(a)) indicated that a short-patch mode of gene conversion (nonreciprocal HR), representing localized strand removal and resynthesis, had created multiple discontinuous tracts during recombination. Even greater complexity was observed in cells transformed with dsDNA that was itself a preformed heteroduplex in which both strands were distinguished from each other as well as from the recipient chromosome (Figure 3(b)). The patterns from these triallelic crosses suggested that the preformed heteroduplex was processed* via* similar localized strand removal and resynthesis before synapsis with the recipient chromosome [48].

## 10. Could Fork Regression Target Replication Errors for Removal?

**Figure 4:**
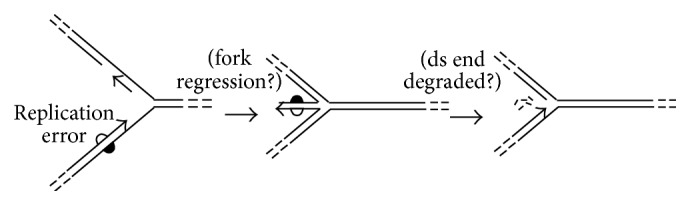
Repositioning of replication errors by fork reversal. If a replication fork reverses shortly after making a replication error, the resulting mismatch would be localized to the short (extruded) arm. In principle, this could provide a basis for removing replication errors without involving a conventional (MutSL-dependent) MMR system. The white semicircle represents a polymerase error; heavy lines depict parental (template) strands.

Transient reversal of the replication fork to form a four-arm structure, commonly termed a “chicken foot,” is thought to be relatively frequent in DNA replication and is proposed to have functional roles, such as providing error-free alternatives to TLS when the leading-strand template has an unrepaired lesion [49, 50]. One property of regressed forks that has not received much attention is the distinctive nature of the fourth (i.e., extruded) arm, which (i) represents the only DNA end of the structure (albeit not necessarily a blunt one) and (ii) includes only the newly synthesized daughter strands (Figure 4). Thus, a “chicken foot” contains, in the configuration of its strands, the information needed to find and remove a recent replication error (Figure 4). Although this hypothetical “spellchecker” would remove a correct strand along with the replication error, this price may be considered small in light of the potential simplicity of the mechanism (which does not need to track strand discontinuities) and the benefits of the resulting replication accuracy. However, this hypothetical mode of strand discrimination would impose some unusual and perhaps unrealistic requirements. The replication fork should presumably reverse only when a replication error has left either of the DNA polymerases, for example; also, destruction of the fourth arm would need to be prevented in forks reversed in the course of other processes, such as error-free lesion bypass [50].

## 11. The Enigma of Essential Recombination

Recombination proteins play diverse roles across biology. Historically, analysis of homologous recombination (HR) emphasized genetic diversification, that is, the reassortment of alleles in eukaryotic meiosis and the acquisition of new traits in bacteria following chromosomal DNA transfer. Increasingly, however, HR is considered to support successful genome replication by providing repair of double-strand breaks (DSBs). In particular, the central process, invasion of a DNA duplex by a single-stranded 3′ end with a matching sequence, provides a route to reassembling broken replication forks [37, 38].

HR in bacteria can be eliminated nearly quantitatively by inactivating the homolog of the* E. coli recA* gene or in eukaryotes by inactivating the Rad52 protein, which loads the eukaryotic equivalent of RecA (Rad51) onto ssDNA. Archaea have their own recombinases, designated “RadA,” related to both the bacterial and eukaryotic proteins [51]. All these proteins mediate a central step in HR, in which ssDNA finds its complement in dsDNA and pairs with it. In addition to abolishing the processes of crossing-over and gene conversion between two homologous duplex DNAs,* recA* or* rad52* mutations make cells dramatically sensitive to DNA damage, and this sensitivity is exacerbated if NER is also disabled. This supports the argument that the immediate, and perhaps the original, function of HR is to enable replication forks blocked by unrepaired lesions to disassemble, reform, and resume DNA replication [39, 52].

The remaining proteins required for HR prepare the DNA end(s) for strand invasion or resolve the structures that result. In bacteria, homologs of the* E. coli* RecBCD proteins prepare the ds end for invasion of the intact duplex. The RecBCD complex is a powerful helicase/exonuclease that initially unwinds and degrades both strands of dsDNA end but eventually converts to a form that leaves 3′ extensions, which are needed for strand invasion [37, 38]. Another complex in bacteria, consisting of the SbcCD proteins, can also perform this function, and eukaryotic cells have corresponding “MRX” complexes, composed of related eukaryotic proteins Mre11 and Rad50 (the third protein, X, differs among different eukaryotes). Archaea have their own version of MRX, composed of archaeal Mre11 and Rad50 homologs, and a uniquely archaeal complex of bipolar helicase and endo/exonuclease, HerA-NurA [53].

Among archaea, genetic dissection of HR functions has made the most progress in the extreme halophiles. The* radA*,* rad50*, and* mre11* genes have been deleted individually and in combinations from* Halobacterium salinarum* and* Haloferax volcanii*, for example, [54–56]. Most of the mutants showed a modest increase in radiation sensitivity, but in* Haloferax*,* radA* could not be deleted from a mutant lacking all four of the normal replication origins [57]. In contrast to the halophiles, HA have not permitted deletion of the* radA* gene, despite multiple attempts by multiple groups. Furthermore, four other proteins of HA share this property of apparent essentiality: the archaeal homologs of Mre11 and Rad50, as well as the HerA and NurA proteins. All of these proteins are implicated in the central steps of HR, that is, the processing of dsDNA and for RadA loading and subsequent strand invasion, and to the author's knowledge they have not been identified with any other, nonrecombination, function. The* mre11*,* rad50*,* herA*, and* nurA* genes are typically clustered in HA genomes, and the proteins have been observed to form foci in *γ*-irradiated cells [58]. The apparent essentiality of these proteins therefore seems to coincide with the other genetic features discussed above that distinguish HA from all other cellular organisms, including mesophilic archaea.

## 12. Do HA Need HR to Survive Fork-Centered DNA Repair?

In principle, the essentiality of RadA, Mre11, Rad50, HerA, and NurA specifically in HA could reflect biochemical functions unique to the HA versions of these proteins. Although this possibility remains intriguing, it seems more parsimonious to ask whether the broadly conserved function of these proteins (specifically, HR between a dsDNA end and a corresponding duplex) is not simply in greater demand or plays a more critical role in HA than in other organisms. If HA indeed lack a dedicated, preventive-maintenance system like NER to repair bulky, helix-distorting lesions before replication, they should experience frequent lesion-induced fork stalling. Bacterial and eukaryotic cells experience such arrest, and HR proteins are required for many of their responses to this challenge [39, 52]. In HA, therefore (i) the ability to remove UV lesions from genomic DNA in the absence of conventional NER [34], (ii) the importance of Hef/Xpf activity for full DNA repair capacity [36], and (iii) the essentiality of basic HR functions for viability (Table 1) combine to suggest the possibility that breakage and HR-mediated reassembly of replication forks blocked at DNA lesions may serve as the major pathway for removing such lesions.

**Figure 5:**
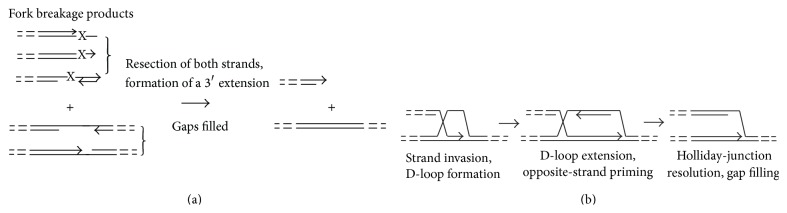
Regeneration of broken replication forks by HR functions. (a) The products of archaeal replication fork breakage proposed in Figure 2 are expected to retain various lesions, gaps, and overhangs; possible structures are illustrated to the left of each bracket. These various structures must be converted to a “clean” 3′ extension on the ds end and an intact continuous duplex with which it can recombine. (b) Proteins of double-strand-break repair (homologous recombination) promote subsequent reassembly of the replication fork. The steps depicted here are those commonly proposed for bacteria and eukaryotes [37, 38, 52]. As in previous figures, broken lines indicate the remainder of the chromosome, and arrowheads mark 3′ ends.

Most schemes of fork “collapse” (breakage) and reassembly that have been proposed for bacteria or eukaryotes either restore the context of the original lesion, so that it can be repaired by conventional NER, or leave it behind in an associated daughter-strand gap [39, 52]. In the context of HA, it would seem to benefit the cell more if the endonucleolytic cleavage that breaks the replication fork would leave the lesion near the ds end (Figure 4). The proposed advantage of this feature is that the unwinding and bidirectional nucleolytic activities typical of DSB end-processing complexes seem well suited to remove a broad spectrum of DNA lesions, including clustered and opposed ones, with minimal requirements for damage recognition (Figure 5). Similarly, by analogy to the RecBCD complex, once a bidirectional strand removal has discarded the lesion, limiting or inhibiting the 3′-specific activity would, in principle, be sufficient to generate the 3′ tail required to initiate fork reconstruction.

Regardless of its molecular details, the basic principle that all replication-blocking DNA lesions may be funneled down a pathway culminating in fork breakage can be expected to make successful replication of the chromosome dependent on HR to a degree not represented in any other major group of organisms. Although NER mutants of bacteria or eukaryotes manage to reproduce under laboratory conditions on a basis similar to that proposed here for wild-type HA, the envisaged archaeal strategy seems unlikely to succeed in nature without molecular adaptations that make the requisite fork-breakage and reassembly processes unusually efficient and reliable. Genes or biochemical functions found to be unexpectedly important for normal chromosomal replication in HA may include those that embody such adaptations.

Finally, whether or not any of the processes proposed here ultimately prove to operate in HA, the prospect remains that these organisms may integrate repair, recombination, and replication of DNA much more tightly than any other type of cell that has been examined previously. Such integration is expected to make functional studies challenging and anticipates a need for genetic techniques such as construction of single-gene knockout libraries and conditional mutants, epistasis testing, and analysis of mutants with biophysical methods and the full spectrum of molecular biology techniques. Although activities and molecular interactions of individual enzymes will continue to be identified in HA that have a plausible connection with DNA repair and replication fidelity, the most meaningful test of progress in this area will be whether the proposed contribution to genome maintenance can be confirmed in living archaeal cells.

## Figures and Tables

**Table 1:** Gene-deletion studies in hyperthermophilic archaea.

Gene	ORF ID	Organism	Predicted function	Phenotype	Reference
Group I (relevant phenotype)					
*udg2 *	SiRe_0084	*S. islandicus *	Uracil DNA glycosylase	Impaired growth	[10]
*udg4 *	SiRe_1884	*S. islandicus *	Uracil DNA glycosylase	Impaired growth	[10]
*hef/xpf *	TK1021	*Tc. kodakarensis *	Structure-specific endonuclease	General damage sensitivity	[34]
*hjm/hel*308	TK1332	*Tc. kodakarensis *	DNA helicase	Sensitive to mitomycin C	[34]
*phr *	Saci_1227	*S. acidocaldarius *	DNA photolyase	Loss of photoreactivation	[59]
*dbh *	Saci_0554	*S. acidocaldarius *	Y-family DNA polymerase	Increased G:C to T:A	[11]
*radC*1	SiRe_0240	*S. islandicus *	RadA paralog	Increased damage sensitivity	[60]
*phrB *	SiRe_0261	*S. islandicus *	DNA photolyase	Photorepair deficiency	[10]

Group I′ (possible phenotype)					
*xpf *	SiRe_1280	*S. islandicus *	3′-flap endonuclease	Slow DNA replication?	[10]
*dpo4 *		*S. solfataricus *	Y-family DNA polymerase	Possible sensitivity to cisplatin	[61]
*xpb *	TK0928	*Tc. kodakarensis *	NER-related helicase	Marginal damage sensitivity	[36]
*xpd *	TK0784	*Tc. kodakarensis *	NER-related helicase	Marginal damage sensitivity	[36]

Group II (no observed impact)					
*xpd *	SiRe_1685	*S. islandicus *	NER-related helicase	None observed	[10]
*xpb1 *	SiRe_1128	*S. islandicus *	NER-related helicase	None observed	[10]
*xpb2 *	SiRe_1526	*S. islandicus *	NER-related helicase	None observed	[10]
*bax1 *	SiRe_1524	*S. islandicus *	Nuclease	None observed	[10]
*uvde *	Saci_1096	*S. acidocaldarius *	Putative UV endonuclease	No effect on UV survival	[59]
*hjc *	TK1175	*Tc. kodakarensis *	Holliday-junction resolvase	None observed	[36]
*hjc *	SiRe_1431	*S. islandicus *	Holliday-junction resolvase	None observed	[10]
*hje *	SiRe_930	*S. islandicus *	Holliday-junction resolvase	None observed	[10]

Group II′ (synthetic lethality)					
*hje* + *hjc *	SiRe_930 & 1431	*S. islandicus *	Holliday-junction resolvases	Double mutant is lethal	[62]

Group III (presumed lethal)					
*radA *	TK1899	*Tc. kodakarensis *	Recombinase	Lethal (not recovered)	[36]
*rad50 *	TK2211	*Tc. kodakarensis *	DSB end-processing	Lethal (not recovered)	[36]
*mre11 *	TK2212	*Tc. kodakarensis *	DSB end-processing	Lethal (not recovered)	[36]
*herA *	TK2213	*Tc. kodakarensis *	DSB end-processing	Lethal (not recovered)	[36]
*nurA *	TK2210	*Tc. kodakarensis *	DSB end-processing	Lethal (not recovered)	[36]
*xpg*/*fen1 *	TK1281	*Tc. kodakarensis *	5′-flap endonuclease	Lethal (not recovered)	[36]
*radA *	SiRe_1747	*S. islandicus *	Recombinase	Lethal	[10]
*herA *	SiRe_0064	*S. islandicus *	Bipolar helicase	Confirmed lethal	[62, 63]
*xpg*/*fen1 *	SiRe_1830	*S. islandicus *	5′-flap endonuclease	Lethal	[10]
*nurA *	SiRe_0061	*S. islandicus *	DSB end-processing	Confirmed lethal	[10, 62]
*rad50 *	SiRe_0062	*S. islandicus *	DSB end-processing	Confirmed lethal	[10, 62]
*mre11 *	SiRe_0063	*S. islandicus *	DSB end-processing	Confirmed lethal	[10, 62]
*hjm *	SiRe_0250	*S. islandicus *	DSB end-processing	Lethal	[62, 64]

## References

[1] Friedberg E. C., Walker G. C., Siede W., Wood R. D., Schultz R. A., Ellenberger T. (2006). *DNA Repair and Mutagenesis*.

[2] Woese C. R., Kandler O., Wheelis M. L. (1990). Towards a natural system of organisms: proposal for the domains Archaea, Bacteria, and Eucarya. *Proceedings of the National Academy of Sciences of the United States of America*.

[3] Barry E. R., Bell S. D. (2006). DNA replication in the archaea. *Microbiology and Molecular Biology Reviews*.

[4] Ishino S., Kelman L. M., Kelman Z., Ishino Y. (2014). The archaeal DNA replication machinery: past, present and future. *Genes and Genetic Systems*.

[5] Kelman L. M., Kelman Z. (2014). Archaeal DNA replication. *Annual Review of Genetics*.

[6] O'Donnell M., Langston L., Stillman B. (2013). Principles and concepts of DNA replication in bacteria, archaea, and eukarya. *Cold Spring Harbor Perspectives in Biology*.

[7] Sarmiento F., Long F., Cann I., Whitman W. B. (2014). Diversity of the DNA replication system in the archaea domain. *Archaea*.

[8] Atomi H., Matsumi R., Imanaka T. (2004). Reverse gyrase is not a prerequisite for hyperthermophilic life. *Journal of Bacteriology*.

[9] Forterre P. (2002). A hot story from comparative genomics: reverse gyrase is the only hyperthermophile-specific protein. *Trends in Genetics*.

[10] Zhang C., Tian B., Li S. (2013). Genetic manipulation in *Sulfolobus islandicus* and functional analysis of DNA repair genes. *Biochemical Society Transactions*.

[11] Sakofsky C. J., Foster P. L., Grogan D. W. (2012). Roles of the Y-family DNA polymerase Dbh in accurate replication of the *Sulfolobus* genome at high temperature. *DNA Repair*.

[12] Huang Q., Li Y., Zeng C. (2015). Genetic analysis of the Holliday junction resolvases Hje and Hjc in *Sulfolobus islandicus*. *Extremophiles*.

[13] Scharer O. D. (2013). Nucleotide excision repair in eukaryotes. *Cold Spring Harbor Perspectives in Biology*.

[14] Lasken R. S., Schuster D. M., Rashtchian A. (1996). Archaebacterial DNA polymerases tightly bind uracil-containing DNA. *The Journal of Biological Chemistry*.

[15] Liu X.-P., Liu J.-H. (2011). Characterization of family IV UDG from *Aeropyrum pernix* and its application in hot-start PCR by family B DNA polymerase. *PLoS ONE*.

[16] Hogrefe H. H., Hansen C. J., Scott B. R., Nielson K. B. (2002). Archaeal dUTPase enhances PCR amplifications with archaeal DNA polymerases by preventing dUTP incorporation. *Proceedings of the National Academy of Sciences of the United States of America*.

[17] Grúz P., Shimizu M., Pisani F. M., de Felice M., Kanke Y., Nohmi T. (2003). Processing of DNA lesions by archaeal DNA polymerases from *Sulfolobus solfataricus*. *Nucleic Acids Research*.

[18] Gaidamaviciute E., Tauraite D., Gagilas J., Lagunavicius A. (2010). Site-directed chemical modification of archaeal *Thermococcus litoralis* Sh1B DNA polymerase: acquired ability to read through template-strand uracils. *Biochimica et Biophysica Acta*.

[19] Jozwiakowski S. K., Keith B. J., Gilroy L., Doherty A. J., Connolly B. A. (2014). An archaeal family-B DNA polymerase variant able to replicate past DNA damage: occurrence of replicative and translesion synthesis polymerases within the B family. *Nucleic Acids Research*.

[20] Wardle J., Burgers P. M. J., Cann I. K. O. (2008). Uracil recognition by replicative DNA polymerases is limited to the archaea, not occurring with bacteria and eukarya. *Nucleic Acids Research*.

[21] Richardson T. T., Gilroy L., Ishino Y., Connolly B. A., Henneke G. (2013). Novel inhibition of archaeal family-D DNA polymerase by uracil. *Nucleic Acids Research*.

[22] Grasso S., Tell G. (2014). Base excision repair in Archaea: back to the future in DNA repair. *DNA Repair*.

[23] Fogg M. J., Pearl L. H., Connolly B. A. (2002). Structural basis for uracil recognition by archaeal family B DNA polymerases. *Nature Structural Biology*.

[24] Jozwiakowski S. K., Borazjani Gholami F., Doherty A. J. (2015). Archaeal replicative primases can perform translesion DNA synthesis. *Proceedings of the National Academy of Sciences of the United States of America*.

[25] Sakai T., Tokishita S.-I., Mochizuki K., Motomiya A., Yamagata H., Ohta T. (2008). Mutagenesis of uracil-DNA glycosylase deficient mutants of the extremely thermophilic eubacterium *Thermus thermophilus*. *DNA Repair*.

[26] Acharya S., Foster P. L., Brooks P., Fishel R. (2003). The coordinated functions of the *E. coli* MutS and MutL proteins in mismatch repair. *Molecular Cell*.

[27] Li G. M. (2008). Mechanisms and functions of DNA mismatch repair. *Cell Research*.

[28] Surtees J. A., Argueso J. L., Alani E. (2004). Mismatch repair proteins: key regulators of genetic recombination. *Cytogenetic and Genome Research*.

[29] Berkner S., Wlodkowski A., Albers S.-V., Lipps G. (2010). Inducible and constitutive promoters for genetic systems in *Sulfolobus acidocaldarius*. *Extremophiles*.

[30] Ly V., Hatherell A., Kim E., Chan A., Belmonte M. F., Schroeder D. F. (2013). Interactions between *Arabidopsis* DNA repair genes UVH6, DDB1A, and DDB2 during abiotic stress tolerance and floral development. *Plant Science*.

[31] Ogrunc M., Becker D. F., Ragsdale S. W., Sancar A. (1998). Nucleotide excision repair in the third kingdom. *Journal of Bacteriology*.

[32] Crowley D. J., Boubriak I., Berquist B. R. (2006). The uvrA, uvrB and uvrC genes are required for repair of ultraviolet light induced DNA photoproducts in *Halobacterium* sp. NRC-1. *Saline Systems*.

[33] White M. F., Garrett R. A., Klenk H. P. (2007). DNA repair. *Archaea: Evolution, Physiology, and Molecular Biology*.

[34] Dorazi R., Götz D., Munro S., Bernander R., White M. F. (2007). Equal rates of repair of DNA photoproducts in transcribed and non-transcribed strands in *Sulfolobus solfataricus*. *Molecular Microbiology*.

[35] Romano V., Napoli A., Salerno V., Valenti A., Rossi M., Ciaramella M. (2007). Lack of strand-specific repair of UV-induced DNA lesions in three genes of the archaeon *Sulfolobus solfataricus*. *Journal of Molecular Biology*.

[36] Fujikane R., Ishino S., Ishino Y., Forterre P. (2010). Genetic analysis of DNA repair in the hyperthermophilic archaeon, Thermococcus kodakaraensis. *Genes and Genetic Systems*.

[37] Forsburg S. L. (2008). The MCM helicase: linking checkpoints to the replication fork. *Biochemical Society Transactions*.

[38] Cox M. M. (2001). Recombinational DNA repair of damaged replication forks in *Escherichia coli*: questions. *Annual Review of Genetics*.

[39] Cox M. M., Goodman M. F., Kreuzer K. N., Sherratt D. J., Sandler S. J., Marians K. J. (2000). The importance of repairing stalled replication forks. *Nature*.

[40] Dillingham M. S., Spies M., Kowalczykowski S. C. (2003). RecBCD enzyme is a bipolar DNA helicase. *Nature*.

[41] Yeeles J. T. P., Poli J., Marians K. J., Pasero P. (2013). Rescuing stalled or damaged replication forks. *Cold Spring Harbor Perspectives in Biology*.

[42] Fitz-Gibbon S. T., Ladner H., Kim U.-J., Stetter K. O., Simon M. I., Miller J. H. (2002). Genome sequence of the hyperthermophilic crenarchaeon *Pyrobaculum aerophilum*. *Proceedings of the National Academy of Sciences of the United States of America*.

[43] Berkner S., Lipps G. (2008). Mutation and reversion frequencies of different *Sulfolobus* species and strains. *Extremophiles*.

[44] Grogan D. W., Carver G. T., Drake J. W. (2001). Genetic fidelity under harsh conditions: analysis of spontaneous mutation in the thermoacidophilic archaeon *Sulfolobus acidocaldarius*. *Proceedings of the National Academy of Sciences of the United States of America*.

[45] Martusewitsch E., Sensen C. W., Schleper C. (2000). High spontaneous mutation rate in the hyperthermophilic archaeon *Sulfolobus solfataricus* is mediated by transposable elements. *Journal of Bacteriology*.

[46] Pata J. D. (2010). Structural diversity of the Y-family DNA polymerases. *Biochimica et Biophysica Acta—Proteins and Proteomics*.

[47] Potapova O., Grindley N. D. F., Joyce C. M. (2002). The mutational specificity of the Dbh lesion bypass polymerase and its implications. *The Journal of Biological Chemistry*.

[48] Mao D., Grogan D. W. (2012). Heteroduplex formation, mismatch resolution, and genetic sectoring during homologous recombination in the hyperthermophilic archaeon *Sulfolobus acidocaldarius*. *Frontiers in Microbiology*.

[49] Atkinson J., McGlynn P. (2009). Replication fork reversal and the maintenance of genome stability. *Nucleic Acids Research*.

[50] Sale J. E. (2012). Competition, collaboration and coordination—determining how cells bypass DNA damage. *Journal of Cell Science*.

[51] Seitz E. M., Brockman J. P., Sandler S. J., Clark A. J., Kowalczykowski S. C. (1998). RadA protein is an archaeal RecA protein homolog that catalyzes DNA strand exchange. *Genes and Development*.

[52] Kuzminov A. (2001). DNA replication meets genetic exchange: chromosomal damage and its repair by homologous recombination. *Proceedings of the National Academy of Sciences of the United States of America*.

[53] Rzechorzek N. J., Blackwood J. K., Bray S. M., Maman J. D., Pellegrini L., Robinson N. P. (2014). Structure of the hexameric HerA ATPase reveals a mechanism of translocation-coupled DNA-end processing in archaea. *Nature Communications*.

[54] Delmas S., Duggin I. G., Allers T. (2013). DNA damage induces nucleoid compaction via the Mre11-Rad50 complex in the archaeon *Haloferax volcanii*. *Molecular Microbiology*.

[55] Kish A., DiRuggiero J. (2008). Rad50 is not essential for the Mre11-dependent repair of DNA double-strand breaks in *Halobacterium* sp. strain NRC-1. *Journal of Bacteriology*.

[56] Woods W. G., Dyall-Smith M. L. (1997). Construction and analysis of a recombination-deficient (*radA*) mutant of *Haloferax volcanii*. *Molecular Microbiology*.

[57] Hawkins M., Malla S., Blythe M. J., Nieduszynski C. A., Allers T. (2013). Accelerated growth in the absence of DNA replication origins. *Nature*.

[58] Quaiser A., Constantinesco F., White M. F., Forterre P., Elie C. (2008). The Mre11 protein interacts with both Rad50 and the HerA bipolar helicase and is recruited to DNA following gamma irradiation in the archaeon *Sulfolobus acidocaldarius*. *BMC Molecular Biology*.

[59] Sakofsky C. J., Runck L. A., Grogan D. W. (2011). *Sulfolobus* mutants, generated via PCR products, which lack putative enzymes of UV photoproduct repair. *Archaea*.

[60] Liang P.-J., Han W.-Y., Huang Q.-H. (2013). Knockouts of RecA-like proteins RadC1 and RadC2 have distinct responses to DNA damage agents in *Sulfolobus islandicus*. *Journal of Genetics and Genomics*.

[61] Wong J. H. Y., Brown J. A., Suo Z., Blum P., Nohmi T., Ling H. (2010). Structural insight into dynamic bypass of the major cisplatin-DNA adduct by Y-family polymerase Dpo4. *The EMBO Journal*.

[62] Huang Q., Liu L., Liu J., Ni J., She Q., Shen Y. (2015). Efficient 5′-3′ DNA end resection by HerA and NurA is essential for cell viability in the crenarchaeon *Sulfolobus islandicus*. *BMC Molecular Biology*.

[63] Zheng T., Huang Q., Zhang C., Ni J., She Q., Shen Y. (2012). Development of a simvastatin selection marker for a hyperthermophilic acidophile, *Sulfolobus islandicus*. *Applied and Environmental Microbiology*.

[64] Hong Y., Chu M., Li Y. (2012). Dissection of the functional domains of an archaeal Holliday junction helicase. *DNA Repair*.

[65] Roberts J. A., Bell S. D., White M. F. (2003). An archaeal XPF repair endonuclease dependent on a heterotrimeric PCNA. *Molecular Microbiology*.

[66] Kaiser M. W., Lyamicheva N., Ma W. (1999). A comparison of eubacterial and archaeal structure-specific 5′-exonucleases. *The Journal of Biological Chemistry*.

[67] Rockwood J., Mao D., Grogan D. W. (2013). Homologous recombination in the archaeon *Sulfolobus acidocaldarius*: effects of DNA substrates and mechanistic implications. *Microbiology*.

